# B Vitamins in Legume Ingredients and Their Retention in High Moisture Extrusion

**DOI:** 10.3390/foods13050637

**Published:** 2024-02-20

**Authors:** Aino Siitonen, Faisa Nieminen, Veronika Kallio, Fabio Tuccillo, Katja Kantanen, Jose Martin Ramos-Diaz, Kirsi Jouppila, Vieno Piironen, Susanna Kariluoto, Minnamari Edelmann

**Affiliations:** Department of Food and Nutrition, University of Helsinki, Agnes Sjöbergin katu 2, FI-00014 Helsinki, Finland; aino.siitonen@helsinki.fi (A.S.); faisanieminen@gmail.com (F.N.); veronika.kallio89@gmail.com (V.K.); fabio.tuccillo@helsinki.fi (F.T.); katja.kantanen@helsinki.fi (K.K.); martin.ramosdiaz@luke.fi (J.M.R.-D.); kirsi.jouppila@helsinki.fi (K.J.); vieno.piironen@helsinki.fi (V.P.); susanna.kariluoto@helsinki.fi (S.K.)

**Keywords:** B vitamin, folate, high moisture extrusion, legume, niacin, retention, riboflavin, thiamin

## Abstract

Legumes have been recognised as healthy and environmentally friendly protein sources. Knowledge about the vitamin B contents in legume ingredients and extrudates is scarce. In this study, we investigated thiamin, riboflavin, niacin, and folate in various faba bean, lupin, and pea ingredients. Further, the retention of B vitamins in high moisture extrusion was studied. Prior to liquid chromatographic determinations of thiamin, riboflavin, niacin, and folate, vitamins were extracted by acid hydrolysis (niacin), enzymatic treatment (folate), or their combination (thiamin and riboflavin). The contents (on a dry matter basis) varied greatly among different ingredients: the thiamin content was 0.2–14.2 µg/g; riboflavin, 0.3–5.9 µg/g; niacin, 8.8–35.5 µg/g, and folate, 45–1453 ng/g. Generally, the highest levels were in flours and protein concentrates, whereas low levels were observed in isolates. The retention of B vitamins was excellent in high moisture extrusion, except for folate in faba bean, where the folate contents were 42–67% lower in the extrudates than in the respective ingredient mixtures. In terms of both vitamin B contents and their retention, extrudates containing substantial amounts of flour or protein concentrate are promising plant-based sources of thiamin, riboflavin, niacin, and folate.

## 1. Introduction

The production of plant-based foods causes a lower environmental impact than the production of animal-based foods [1]. Therefore, alternatives to animal proteins can facilitate a shift towards a more sustainable food system and further ensure food security for the increasing world population [2]. Legumes are superior raw materials for this transition due to their high protein content, which can be up to 30% on a dry-weight basis. Legumes are also a rich source of dietary fibre and several micronutrients [3]. However, they also contain harmful compounds, such as phytic acid, lectins, saponins, and galacto-oligosaccharides [2,4], which pose some limitations on their food use. Nevertheless, due to the nutritional composition of legumes, their regular consumption has been associated with various health benefits, such as improved glycemic control and blood lipid profile [4].

B vitamins are water-soluble compounds that act in the metabolism of carbohydrates, amino and fatty acids, and nucleotides. Legumes have been reported to contain B vitamins, such as thiamin, riboflavin, niacin, and folate [5,6,7]. Nevertheless, animal-based foods are currently important sources of several B vitamins [8]. For example, in the Finnish diet, dairy and meat products contribute 40–60% of the dietary intake of thiamin, riboflavin, and niacin [9]. Folate is an exception: its main sources are cereal products and vegetables. The intake of riboflavin and niacin from a plant-based diet may be insufficient [10], and it is therefore essential to study the B vitamins in plant-based foods.

Legume-derived protein-enriched ingredients—protein concentrates and protein isolates—are produced for food applications to improve techno-functional properties or obtain a high protein content in the final product. These ingredients can be produced using various processing methods [11]. For instance, dry fractionation based on air classification has been applied to obtain protein concentrates, and aqueous extraction followed by isoelectric precipitation or ultrafiltration can be used to produce protein isolates. The obtained protein content in protein concentrates is around 50–60%, while in protein isolates, it may be even more than 90% [12]. The fractionation processes are expected to alter the composition of minor components, which causes concern about the nutritional quality of legume-derived ingredients. The contents of water-soluble antinutrients in faba bean have been observed to retain or increase the production of protein concentrates and are mainly eliminated during the processing of protein isolates due to the leaching in aqueous extraction [11]. However, to our knowledge, there are no previous studies on vitamin B contents in these ingredients.

In recent years, plant-based meat alternatives have gained popularity. Textured plant protein products have been produced by extrusion from protein concentrates and protein isolates made of various legumes, such as soybean, pea, and faba bean [13,14,15]. Extrusion utilises temperature, shear forces, and pressure to cause conformational changes and molecular interactions in extruded material, which leads to the structure formation of extrudates [12]. The formation of structure is affected by combinations of extrusion parameters, such as feed rate, water content, temperature profile, and screw speed. Low moisture extrusion usually produces a dry porous structure, whereas high moisture extrusion with a long cooling die can form a fibrous meat-resembling structure [12]. Therefore, high moisture extrusion technology offers innovative solutions to the increasing demand for plant-based protein products.

In terms of nutritional quality, extrusion may have beneficial effects, including improved protein and starch digestibility and decreased antinutrient contents [16]. Simultaneously, however, extrusion may cause a loss of vitamins. Several studies have examined the influence of low moisture extrusion on vitamin contents in various matrices, but only limited data are available on B vitamins in legume-based extrudates. Thiamin, riboflavin, and niacin retention in low moisture extrusion of legume-based materials have been reported to range from 50% to 85% [17,18]. Ramos Diaz et al. [19] observed that folate was retained relatively well in low moisture extrusion of corn-based material containing lupin. Nevertheless, to our knowledge, only one published study has examined the retention of B vitamins in high moisture extrusion. Caporgno et al. [13] observed excellent stabilities of thiamin, riboflavin, and niacin in high moisture extrusion of a mixture containing soy protein concentrate and microalgae powder. Overall, it remains unclear how high moisture extrusion of various legume ingredients affects B vitamins; hence, investigations are needed.

This study aimed to investigate the potential of various legume ingredients to provide B vitamins—thiamin, riboflavin, niacin, and folate—in food applications. Further, the objective was to examine the retention of B vitamins in high moisture extrusion and to evaluate legume-based extrudates as sources of thiamin, riboflavin, niacin, and folate. The contents were determined from various commercial faba bean, lupin, and pea ingredients, as well as extrudates produced from faba bean and lupin ingredients. The vitamer distributions of niacin and folate were also investigated. In addition, the simultaneous extraction of thiamin and riboflavin, along with their analysis method, was validated in this study.

## 2. Materials and Methods

### 2.1. Enzymes and Standards

β-Amylase (Megazyme Ltd., Bray, Ireland) and Taka-Diastase (138.42 HUT/g; Pfaltz and Bauer, Waterbury, CT, USA) were used in thiamin and riboflavin analysis, while α-amylase (A9857) and protease (P5147) from Sigma-Aldrich (St. Louis, MO, USA) were used in folate extraction. Additionally, the conjugase enzyme was isolated from hog kidneys in the laboratory, and the activity was tested according to Kariluoto et al. [20]. The standards for analysis of thiamin, riboflavin, and niacin were obtained from Sigma-Aldrich (St. Louis, MO, USA) and included nicotinic acid (≥99.5%, NA), nicotinamide (≥99.5%, NAM), riboflavin (≥98%), riboflavin-5′-phosphate (FMN, sodium salt hydrate), flavin adenine dinucleotide (≥95%, FAD, disodium salt hydrate), thiamin (≥99%, hydrochloride, T4625, MW = 337.27), and thiamin pyrophosphate (≥95%, TPP). Folate standards (6S)-tetrahydrofolate (H_4_folate, sodium salt), (6S)-5-methyltetrahydrofolate (5-CH_3_-H_4_folate, calcium salt), (6R,S)-5,10-methenyltetrahydrofolate (5,10-CH^+^-H_4_folate, hydrochloride), and (6S)-5-formyltetrahydrofolate (5-HCO-H_4_folate, sodium salt) were obtained from Merck & Cie (Schaffhausen, Switzerland), and folic acid (pteroylglutamic acid, PGA) and 10-formylfolic acid (10-HCO-PGA) were obtained from Schircks Laboratories (Jona, Switzerland). The 10-formyldihydrofolate (10-HCO-H_2_folate) was synthesised from 5,10-CH^+^-H_4_folate [20]. The concentration of each standard was confirmed with a respective spectrophotometric method.

### 2.2. Legume Materials and Sample Preparation

The legume ingredients were obtained from faba bean (*Vicia faba*), lupin (*Lupinus angustifolius*), and pea (*Pisum sativum*). These ingredients were commercially available products: faba bean flour (FF; Suomen Viljava Ltd., Helsinki, Finland), faba bean protein concentrates (FPC-1 and FPC-2; Suomen Viljava Ltd., Helsinki, Finland and AGT Food and Ingredients Inc., Regina, SK, Canada), faba bean protein isolate (FPI; AGT Food and Ingredients Inc., Regina, Canada), lupin flour (LF) and lupin protein concentrate (LPC; Frank Food Product, Twello, Netherlands), lupin protein isolate (LPI; Prolupin GmbH, Grimmen, Germany), pea flour (PF), pea protein concentrate (PPC), pea fibre isolate (PFI; VestKorn, Tau, Norway), and pea protein isolates (PPI-1 and PPI-2; Roquette Corp., Lestrem, France, and Emsland Group, Emlichheim, Germany). The samples were stored in the dark at −20 °C until the experiments were performed.

Certified reference materials BCR 487 (lyophilised pig liver powder) and BCR 121 (wholemeal flour) from the Institute for Reference Materials and Measurements (Geel, Belgium) were used to validate the thiamin and riboflavin analysis and for the quality control of folate analysis. A commercial faba bean flour (Suomen Viljava Ltd., Helsinki, Finland) was used as the in-house reference sample for the quality control of thiamin, riboflavin, and niacin analysis methods.

Extrudates were produced from faba bean and lupin ingredients using high moisture extrusion, as described in prior publications by Kantanen et al. [15], Ramos-Diaz et al. [21], and Tuccillo et al. [22]. Faba bean protein concentrates, as well as mixtures of faba bean and lupin ingredients, were extruded under various extrusion conditions (Table 1). Extrusion was conducted with a twin-screw laboratory extruder (Thermo Prism PTW24 Thermo Haake, Polylab System, Karlsruhe, Germany) attached to a long cooling die (flat cooling nozzle FKD75, DIL Deutsches Institut für Lebensmitteltechnik, Quakenbrück, Germany) using different feed water contents, screw speeds, and long cooling die temperatures. The temperature profile of the temperature-controlled zones in the extruder barrel was 25 °C, 40 °C, 80 °C, 100 °C, 120 °C, and 150 °C. The temperature of the seventh zone (die) was 150 °C. Extrudates were grounded using a laboratory-scale homogeniser and then freeze-dried prior to the vitamin analyses. The moisture contents in the ingredients and freeze-dried extrudates were determined according to the oven-drying (103 °C) AACC 44-15A method [23], and vitamin contents were calculated on a dry weight basis.

### 2.3. Thiamin and Riboflavin Analysis

A simultaneous extraction method of thiamin and riboflavin from legume-based matrices and the ultra-high performance liquid chromatography (UHPLC) analysis were validated. The validation was performed in terms of linearity, analytical sensitivity, precision, and recovery. The extraction of thiamin and riboflavin was based on the European standard methods [24,25]. In the extraction, acid hydrolysis and enzymatic dephosphorylation of thiamin and riboflavin were performed simultaneously, and then the vitamins were determined using the UHPLC method. Riboflavin was detected with a fluorescence detector (FLR) as such, but FLR detection of thiamin required pre-column derivatisation. Thiamin contents were expressed as thiamin chloride hydrochloride. The conversion factor to obtain results as thiamin is 0.787.

#### 2.3.1. Extraction and Enzyme Treatment of Thiamin and Riboflavin and Pre-Column Derivatisation of Thiamin

In brief, a homogenised sample (0.5 g) was mixed with 15 mL of 0.1 M HCl, and the pH was adjusted to 2 with 2.5 M sodium acetate. The sample was heat-extracted for 60 min in a boiling water bath. After cooling, the pH was adjusted to 4.5 with 2.5 M sodium acetate, and the extract was subsequently incubated for 16 h at 37 °C with Taka-Diastase (50 mg) and β-amylase (5 mg). Then, centrifugation (8800× *g*, 10 min) was conducted, and the supernatant was paper-filtered into a 25 mL volumetric flask, which was filled to mark with Milli-Q water. Prior to riboflavin analysis with UHPLC, around 1 mL of the sample extract was filtered through a 0.2 µm syringe filter (wwPTFE membrane).

Conversion of thiamin to fluorescent thiochrome derivate was performed by mixing 1 mL of sample extract with 1 mL of alkaline potassium hexacyanoferrate solution. The mixture was vortexed for 20 s, and after standing for 1 min, the reaction was stopped by adding 100 µL of sodium sulphite solution (100 mg/mL). Finally, the sample extract was filtered using a 0.2 µm syringe filter (wwPTFE membrane).

Samples containing faba bean flour and protein concentrate were found to require a purification step prior to derivatisation. Solid phase extraction (SPE) with a weak cation exchange cartridge (Oasis WCX cartridge, 6 mL, 150 mg; Waters Corporation, Milford, MA, USA) was used for purification, as previously described by Gratacós-Cubarsí et al. [26]. Briefly, the cartridge was conditioned with 5 mL of methanol, followed by 5 mL of Milli-Q water. The sample extract (5 mL), which was adjusted to pH 6.0 with 2.5 M sodium acetate, was applied and subsequently washed with 4 mL of Milli-Q water and 4 mL of methanol. Finally, thiamin was eluted with 4 mL of methanol containing 2% formic acid. The eluent was evaporated with nitrogen flow at 25 °C, and the residue was dissolved in Milli-Q water with the pH adjusted to 4.5.

#### 2.3.2. UHPLC Analysis of Thiamin and Riboflavin

The determination of riboflavin and thiamin was performed using a Waters ACQUITY UPLC system (Waters Corporation, Milford, MA, USA) composed of a sample manager, binary solvent manager, column manager, and photodiode array (PDA), as well as FLR detectors. UHPLC analysis was based on the method for riboflavin determination previously described by Chamlagain et al. [27]. Separation was conducted on a BEH C18 column (1.7 µm, 2.1 × 100 mm; Waters Corporation, Milford, MA, USA) separately for riboflavin and thiamin. The column temperature was 30 °C, and the injection volumes of the sample extracts were 5 µL for thiamin and 10 µL for riboflavin. Ammonium acetate (20 mM) dissolved in 30% aqueous methanol was used as a mobile phase with a constant flow rate of 0.2 mL/min. The total run time was 6 min. FLR detection with excitation wavelengths of 365 nm and 432 nm, and emission wavelengths of 435 nm and 520 nm were performed to detect thiamin and riboflavin, respectively. Vitamins were identified based on their retention times and quantified using external calibration curves. The calibration range was 0.03–0.61 ng/injection for thiamin and 0.08–1.99 ng/injection for riboflavin.

#### 2.3.3. Method Validation of Thiamin and Riboflavin Analysis

The linearity of the UHPLC method was tested at calibration ranges of 0.03–0.61 ng/injection for thiamin and 0.08–1.99 ng/injection for riboflavin. The sensitivity of the UHPLC method was determined by the limit of detection (LOD), based on the signal-to-noise ratio of three, and the limit of quantification (LOQ), which was equal to 3-fold LOD. The precision of the UHPLC method was monitored by analysing the intra- and inter-day variabilities. The relative standard deviation (RSD%) was calculated for the retention times and peak areas of thiamin and riboflavin in standard solutions. An in-house reference sample (commercial faba bean flour) was analysed on four different days to evaluate the repeatability of the entire analysis method. The precision and performance of the analysis method were monitored by analysing the certified reference materials BCR 487 (pig liver) and BCR 121 (whole meal flour) several times (*n* = 10). Additionally, the performance of the analysis method was evaluated by spiking faba bean flour with thiamin and riboflavin (2 µg) and determining the recoveries of these standards. The recovery test was conducted in triplicate on two separate analysis days. The recovery test for thiamin included SPE purification. The efficiency of Taka-Diastase with β-amylase to dephosphorylate thiamin and riboflavin vitamers was studied using TPP, FMN, and FAD standards (2 µg), which were extracted and analysed (*n* = 3) as samples, and the recovery of thiamin and riboflavin was calculated.

### 2.4. Niacin Analysis

Acid hydrolysis was performed to extract the available niacin according to the European standard method [28]. The total available niacin content was determined as the sum of niacin vitamers—NA and NAM—using the UHPLC method previously validated by Chamlagain et al. [29]. The method included post-column derivatisation to form fluorescent NA and NAM derivatives.

#### 2.4.1. Extraction of Niacin

A homogenised sample (0.5 g) was mixed with 15 mL of 0.1 M HCl solution and heat-extracted for 60 min in a boiling water bath. After cooling, the pH was adjusted to 4.5 with 2.5 M sodium acetate. The extract was centrifuged (8800× *g*, 10 min) and the supernatant was filtered into a 25 mL volumetric flask, which was filled to mark with Milli-Q water.

#### 2.4.2. UHPLC Analysis of Niacin

The chromatographic analysis of niacin was conducted using a Waters ACQUITY UPLC system (see Section 2.3.2). The separation of niacin vitamers (NA and NAM) was conducted on an HSS T3 C18 column (1.8 µm, 2.1 × 150 mm; Waters Corporation, Milford, MA, USA) at 30 °C with an isocratic elution (0.3 mL/min). The total run time was 15 min, and the injection volume of the sample extract was 15 µL. The composition of the mobile phase was as follows: 70 mM potassium dihydrogen phosphate, 150 mM hydrogen peroxide, and 5 μM copper sulphate. The post-column derivatisations of NA and NAM were applied with UV irradiation (366 nm, 8 W) and with the presence of hydrogen peroxide and copper ions in a knitted polytetrafluoroethylene (PFET) reaction coil (1.59 mm o.d., 0.17 mm i.d., and 5 m in length). Accordingly, NA and NAM were detected by FLR detector (excitation wavelength of 322 nm and emission wavelength of 380 nm) and quantified by external calibration curves with calibration ranges of 0.97–48.5 ng/injection for NA and 1.03–51.3 ng/injection for NAM.

The detector responses for NA and NAM were lower than in a previous study conducted in our laboratory [29]. Therefore, the lowest point of the calibration curve was considered to be LOQ for both vitamers (3 µg/g). The inter-day linearity of the calibration curves and peak areas of NA and NAM in individual standard injections were compared to monitor the performance and repeatability of the UHPLC analysis. In addition, because a certified reference material for niacin determination was not available, a commercial faba bean flour was analysed several times (*n* = 10) for niacin and used to ensure the performance and repeatability of the entire method of analysis. The niacin content of the in-house reference sample was 18.5 ± 1.4 µg/g (RSD: 7.5%).

### 2.5. Folate Analysis

Folate vitamers were extracted using a tri-enzyme treatment with α-amylase, hog kidney conjugase, and protease as previously described by Edelmann et al. [30]. Total folate content was determined as a sum of the vitamers—H_4_folate, 5-CH_3_-H_4_folate, 5-HCO-H_4_folate, PGA, 10-HCO-PGA, and 5,10-CH^+^-H_4_folate—using the UHPLC method validated by Liu et al. [31]. 

#### 2.5.1. Folate Extraction, Enzyme Treatment, and Purification

In brief, a homogenised sample (1 g) was boiled for 10 min in a water bath with 12 mL of buffer (50 mM CHES, 50 mM HEPES, 2% sodium ascorbate, and 10 mM 2,3-dimercapto-1-propanol in water; pH 7.85). After cooling, the extract (pH 4.9) was incubated for 3 h at 37 °C with α-amylase (20 mg) and hog kidney conjugase (1 mL of enzyme solution). Then, the extract (pH 7) was incubated for 60 min at 37 °C with protease (8 mg). The enzymes were inactivated by boiling the extract for 5 min. After cooling, the samples were centrifuged (8800× *g*, 10 min), and the supernatant was filtered into a 25 mL volumetric flask. The filtered extract (10–15 mL) was purified and concentrated by affinity chromatography with affinity agarose gel (Affi-Gel 10; Bio-Rad Laboratories, Richmond, CA, USA) and folate-binding protein (Scripps Laboratories, San Diego, CA, USA), as previously explained by Edelmann et al. [30].

#### 2.5.2. UHPLC Analysis of Folate

Determination of the monoglutamate forms of folate vitamers was performed using the Waters ACQUITY UPLC system (see Section 2.3.2). The vitamers were separated on a Kinetex PS C18 column (2.6 µm, 2.1 × 150 mm; Phenomenex, Torrance, CA, USA) at 30 °C. The injection volume of the sample extract was 35 µL, and the mobile phase consisted of 0.7% formic acid in water and in acetonitrile. The gradient elution was performed with a flow rate of 0.6 mL/min, and the proportion of 0.7% formic acid in acetonitrile gradually increased from 5% to 80%. The total run time was 8 min. The detection of folate vitamers was carried out using the PDA detector (290 nm for 10-HCO-H_2_folate, PGA, and 5-HCO-H_4_folate; 360 nm for 5,10-CH^+^-H_4_folate) and the FLR detector (excitation wavelength of 290 nm and emission wavelength of 356 nm for H_4_folate and 5-CH_3_-H_4_folate; excitation wavelength of 360 nm and emission wavelength of 460 nm for 10-HCO-PGA). Folate vitamers were quantified using external calibration curves (0.08–1.44 ng/injection, except for 5-HCO-H_4_folate, 5,10-CH^+^-H_4_folate, and 10-HCO-H_2_folate: 0.32–1.44, 0.16–1.44, and 0.16–2.88 ng/injection, respectively). To monitor the performance of the UHPLC analysis, peak areas of the folate vitamers in individual standard injections were compared on different analysis days. Additionally, the performance of the entire analysis method was verified by analysing the certified reference material BCR 121 with a total folate content of 441 ng/g on a dry weight basis, which was in agreement with the certified value.

### 2.6. Calculation and Statistical Analysis

Vitamin analyses (extraction and further determination) were conducted in triplicate (*n* = 3); the contents were expressed as mean ± standard deviation (SD) on a dry matter (DM) basis. The total folate and niacin contents were calculated as the sum of the respective vitamers. A blank sample (water) was analysed, and the vitamin contents in actual samples were corrected for the possible vitamin contents derived from the enzymes used in the analysis. One-way analysis of variance (ANOVA) and Tukey’s honestly significant difference post hoc test (using IBM SPSS Statistics 22; IBM Corp., Armonk, NY, USA) were applied to compare the vitamin contents of various ingredients. An independent sample *t*-test was used to evaluate the differences in vitamin contents between the ingredient mixtures and corresponding extrudates. A difference was considered to be statistically significant with a *p*-value < 0.05.

## 3. Results and Discussion

### 3.1. Method Performance of Thiamin and Riboflavin Analysis

The method validation results are shown in Table 2. The linearity was excellent at calibration ranges (R^2^ > 0.99), ranging from 0.03 ng/injection to 0.61 ng/injection for thiamin and 0.08 ng/injection to 1.99 ng/injection for riboflavin. Thiamin had an LOD of 2.2 ng/g, and, hence, the LOQ (3 × LOD) was 6.6 ng/g. The LOD and LOQ for riboflavin were 48 ng/g and 144 ng/g, respectively. The results of the intra- and inter-day precision measurements indicated good reproducibility of the UHPLC method. In addition, results for the in-house reference sample (RSD approximately 10%) demonstrated good repeatability of the entire method of analysis. The vitamin contents determined in the certified reference materials BCR 121 and BCR 487 (wholemeal flour: 4.8 ± 0.2 µg/g DM of thiamin; pig liver: 9.8 ± 1.3 µg/g DM of thiamin and 116 ± 4.7 µg/g DM of riboflavin) were in line with the certified values, proving excellent precision and performance of the analysis method. Additionally, the recovery of thiamin and riboflavin in faba bean flour was good (≥85%), and the recoveries of thiamin from TPP (97%) and riboflavin from FMN (76%) and FAD (82%) indicated good dephosphorylating activity of the enzymes.

In this study, we observed interference in the pre-column derivatisation step of thiamin analysis. The conversion of thiamin to thiochrome was incomplete in faba bean flour and protein concentrate extracts. When the extracts were purified with weak cation exchange cartridges prior to the conversion of thiamin to thiochrome, the contents were 1.4-fold to 4-fold higher in the faba bean samples than without purification. In contrast, the purification did not affect the results of faba bean protein isolate, or lupin and pea samples. In conclusion, we emphasise the need for SPE purification in the thiamin extraction procedure of faba bean samples to ensure proper pre-column derivatisation.

### 3.2. B Vitamins of Legume Ingredients

#### 3.2.1. Flours

**Figure 1:**
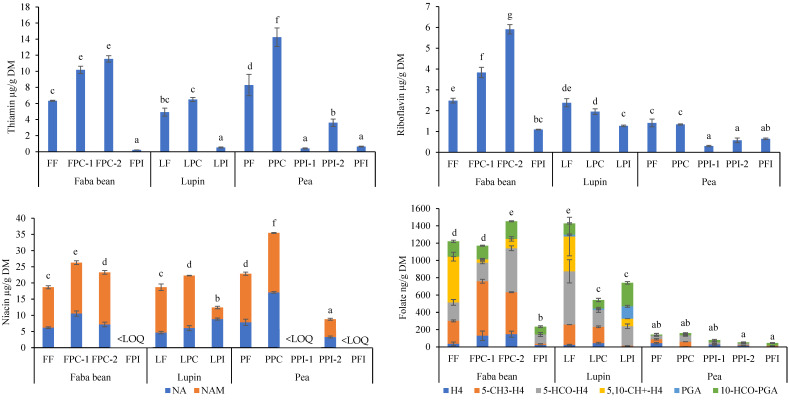
Thiamin, riboflavin, niacin, and folate contents on a dry matter basis (µg/g DM; ng/g DM for folate) in legume ingredients. FF: faba bean flour; FPC: faba bean protein concentrate; FPI: faba bean protein isolate; LF: lupin flour; LOQ: limit of quantification; LPC: lupin protein concentrate; LPI: lupin protein isolate; PF: pea flour; PPC: pea protein concentrate; PPI: pea protein isolate; PFI: pea fibre isolate. Numbers 1 and 2 distinguish different manufacturers. Error bars represent the standard deviation of triplicate determination (*n* = 3), and different letters indicate significant differences (*p* < 0.05) in vitamin content among the ingredients.

Thiamin, riboflavin, and niacin contents in the flours from different legume species were relatively similar, whereas total folate contents varied greatly (Figure 1). The thiamin content in faba bean, lupin, and pea flour ranged from 4.9 µg/g DM to 8.3 µg/g DM, the riboflavin content from 1.4 µg/g DM to 2.5 µg/g DM, and the niacin content from 18.7 µg/g DM to 22.9 µg/g DM. In faba bean and lupin flour, we determined folate content of 1221 ng/g DM and 1428 ng/g DM, respectively. In contrast, folate content was notably lower (142 ng/g DM) in pea flour. Hence, faba bean and lupin flour contained about 9-fold to 10-fold more folate than pea flour.

Knowledge about the vitamin B contents in various legume seeds and commercial legume flours is limited. The vitamin B contents determined in the flours were compared with the existing data regarding their content in legume seeds, although the storage stability of vitamins is most likely better in the initial seeds than in commercial legume flours. Cultivar and growing conditions also have an impact on vitamin content.

Thiamin contents in faba bean, lupin, and pea flours were in the range of contents in legume seeds determined by Witten and Aulrich [7]. They observed that the thiamin content in legume seeds varied with the legume variety, harvest year, and harvest site. The content (converted to thiamin chloride hydrochloride) in faba bean seeds varied from 3.2 µg/g DM to 9.4 µg/g DM, in blue lupin seeds from 3.6 µg/g DM to 11.4 µg/g DM, and in peas from 3.4 µg/g DM to 12.2 µg/g DM. Other authors have also reported comparable thiamin contents in faba bean seeds (4.5 µg/g DM) and white lupin seeds (3.9 µg/g DM) [6,32]. 

The riboflavin content in faba bean seeds has been determined to fall within a range of 2.1 µg/g DM to 3.8 µg/g DM [6,7], which is in accordance with our results. Likewise, in blue lupin seeds, the riboflavin content has been observed to vary from 1.9 µg/g DM to 3.1 µg/g DM, and in peas from 1 µg/g DM to 2.3 µg/g DM [7]. In addition, Erbaş et al. [32] determined a riboflavin content of 2.3 µg/g DM in white lupin seeds.

The determined niacin contents in legume flours represent the available niacin due to the extraction method used. The content in faba bean flour was similar to the value for faba bean seeds (14.5 µg/g DM) reported by Marshall et al. [6]. In contrast, the niacin content in pea and lupin flour was notably lower than the previously reported values for these legumes. The observed niacin content in peas was 37.8 µg/g, and in white lupin seeds, it was 39.1 µg/g DM [5,32]. 

In line with our results, the folate content in faba bean flour and dried seeds has been reported to range from 860 ng/g DM to 1420 ng/g DM [33,34]. Also, a markedly higher folate content in faba bean seeds (2550 ng/g DM) has been observed [6]. A low level of folate (around 100–300 ng/g) in dried peas was observed earlier by Jha et al. [35] and Rychlik et al. [36], which is in accordance with our finding of low folate content in pea flour.

In general, we determined notable vitamin B contents in faba bean, lupin, and pea flours. In addition to comparable values with legume seeds, the contents in legume flours were at approximately the same levels as in cereal grains [37]. Regarding niacin, it should be noted that, in cereal matrices, niacin may occur in both available and bound forms. Therefore, the possible bound forms of niacin in legumes should be studied in the future. Overall, legume flours are promising plant-based sources of thiamin, riboflavin, niacin, and folate.

#### 3.2.2. Protein Concentrates and Isolates

Thiamin, riboflavin, niacin, and folate contents in the legume ingredients varied among the different ingredient types (Figure 1). The vitamin B contents were mainly higher in protein concentrates than in fibre and protein isolates. The only exception was folate in lupin, where concentrates and isolates had similar folate contents. Isolates were generally low in B vitamins. Overall, within the same legume species, the contents of each vitamin in the protein concentrates were mainly similar or even slightly higher than those in flours. As a result, in addition to legume flours, protein concentrates provide a marked amount of B vitamins in food applications.

The thiamin content was consistently the highest in the protein concentrates, followed by flours and isolates, regarding ingredients produced from the same legume species. The contents in protein concentrates ranged from 6.5 µg/g DM to 14.2 µg/g DM and in fibre and protein isolates only from 0.2 µg/g DM to 3.6 µg/g DM. Thiamin contents in the two faba bean protein concentrates from different producers were similar, whereas the two pea protein isolates had significantly (*p* < 0.05) different contents.

Similar to thiamin, the highest levels of riboflavin among faba bean ingredients were found in faba bean protein concentrates (3.8 µg/g DM and 5.9 µg/g DM). In contrast, the riboflavin contents in lupin and pea protein concentrates (2.0 µg/g DM and 1.3 µg/g DM) were similar to that in flours made from the same legume species. Fibre and protein isolate samples had low riboflavin contents (0.3–1.3 µg/g DM). In contrast to thiamin, the riboflavin contents in the two faba bean protein concentrates, which were obtained from different manufacturers, were significantly different (*p* < 0.05), but no difference was observed between the two pea protein isolates.

The niacin content in protein concentrates ranged from 22.3 µg/g DM to 35.5 µg/g DM, and these contents were the highest among different ingredients produced from the same legume species. A significant difference (*p* < 0.05) was observed in niacin contents between the two different faba bean protein concentrates. Among the isolate samples, niacin was quantified only in lupin and one pea protein isolate (12.4 µg/g DM and 8.8 µg/g DM). The contents of NA and NAM in other isolate samples were under the LOQ (< 3 µg/g DM).

Faba bean protein concentrates had high folate contents—1170 ng/g DM and 1453 ng/g DM—whereas a notably lower folate content was determined in faba bean protein isolate (234 ng/g DM). Interestingly, the folate content in the lupin protein isolate (741 ng/g DM) was about the same level as that in the lupin protein concentrate (543 ng/g DM). In addition, all pea ingredients were consistently low in folate (45–160 ng/g DM). The folate contents of the two faba bean protein concentrates produced by different manufacturers were significantly different (*p* < 0.05), whereas the two pea protein isolates had similar contents.

Dry fractionation, which can be used to produce protein concentrates, separates protein-rich and starch-rich fractions of legumes [11]. In turn, the production of protein isolates utilises aqueous extraction, which leads to the leaching of water-soluble compounds. Additionally, the stability of vitamins is always a concern. To our knowledge, the impact of fractionation on vitamins in legumes or the vitamin B contents of protein-enriched legume ingredients has not been previously reported.

In the present study, we compared the vitamin contents of various legume ingredients. Flours and protein concentrates had relatively similar vitamin B contents; in fact, some of the protein concentrates had somewhat higher contents. Hence, vitamins were retained or concentrated in the production of protein concentrates. In contrast, vitamin B contents in both fibre and protein isolates were generally low, in some cases even below the LOQ, proving that the production of isolates caused the loss of B vitamins. Vogelsang-O’Dwyer et al. [11] studied the impact of dry and aqueous fractionation on the contents of water-soluble antinutrients in faba beans and yielded results that agree with our findings. In the production of protein concentrate by dry fractionation, the contents of vicine and convicine were retained, and the content of the raffinose family oligosaccharides increased. Conversely, these contents decreased in the production of protein isolate due to leaching during aqueous extraction.

The ingredients in our study were individual commercial products obtained from different manufacturers, and the exact processing methods used to produce these ingredients were unknown. For this reason, we could not study the direct impact of the fractionation process. However, we can conclude that the B vitamin contents were either retained or even increased in the production of legume protein concentrates, whereas manufacturing fibre and protein isolates caused the loss of water-soluble vitamins.

### 3.3. Vitamer Distributions of Niacin and Folate in Legume Ingredients

Both niacin vitamers—NA and NAM—were observed in all ingredients (Figure 1). Generally, NAM was the main vitamer in the ingredients, contributing 61–75% to niacin content in most of the samples. The distribution of vitamers was nearly equal in the pea protein concentrate, and in the lupin protein isolate, only about 30% of the niacin existed as NAM. The finding that NAM was the main vitamer in legume flours was in accordance with a previous publication. Catak [5] and Ndaw et al. [38] reported that NAM contributed ca. 70–90% to the niacin content in peas.

Four of the folate vitamers—H_4_folate, 5-CH_3_-H_4_folate, 5-HCO-H_4_folate, and 10-HCO-PGA—were found in all ingredients (Figure 1). In addition, 5,10-CH^+^-H_4_folate and PGA were observed in some of the samples. The most abundant folate vitamer in faba bean flour was 5,10-CH^+^-H_4_folate (43%), followed by 5-CH_3_-H_4_folate (22%) and 5-HCO-H_4_folate (17%), which is in line with the findings in faba bean samples reported by Hefni et al. [33] and Liu et al. [34]. The major vitamers in lupin flour were 5-HCO-H_4_folate (43%) and 5,10-CH^+^-H_4_folate (28%). In pea flour, we observed a similar contribution (approximately 30%) of each reduced vitamer (H_4_folate, 5-CH_3_-H_4_folate, 5-HCO-H_4_folate) to the total folate content. Likewise, Jha et al. [35] found that these vitamers were the most abundant in peas. In the protein concentrates, the predominant folate vitamers were consistently 5-CH_3_-H_4_folate (33–54%) and 5-HCO-H_4_folate (18–46%). Therefore, the distribution of endogenous vitamers seems to be retained in the fractionation process of protein concentrates. Conversely, 10-HCO-PGA was one of the main vitamers in several isolate samples, but its proportion was relatively low in flours and protein concentrates. Hence, it seems that this oxidised vitamer was retained or formed in the production of isolates. Accordingly, varying stabilities of folate vitamers including interconversion, degradation, and leaching are plausible reasons for the differing folate vitamer distributions among ingredients.

### 3.4. Retention of B Vitamin in High Moisture Extrusion of Faba Bean and Lupin Ingredients

The vitamin contents in the faba bean and lupin ingredient mixtures and corresponding extrudates varied depending on the proportion of ingredients (Table 3). The temperature of the long cooling die did not consistently or considerably affect the vitamin contents in extrudates. The retentions of thiamin, riboflavin, and niacin were generally good in high moisture extrusion, although some of the thiamin, riboflavin, and niacin contents in extrudates were significantly lower (3–28%; *p* < 0.05) than the contents in the respective ingredient mixtures. However, the differences between the ingredient mixture and corresponding extrudates were relatively minor, and no consistent trend was observed. Interestingly, some of the thiamin and riboflavin contents in the lupin extrudates were higher than the contents in the initial ingredient mixtures. Feasible explanations for this could be that the vitamins were released from their bound forms by extrusion, and the extractability of vitamins from the extrudates was improved as a result.

To our knowledge, only one study has assessed vitamin retention in high moisture extrusion. Caporgno et al. [13] used a mixture of soy protein concentrate and microalgae powder (50:50 DM) in high moisture extrusion with a long cooling die and similar conditions as in our extrusion experiments. They observed excellent stability of thiamin, riboflavin, and niacin, which is in accordance with our findings of retention of these vitamins in the production of faba bean and lupin extrudates. Several studies have investigated the impact of low moisture extrusion on vitamins in cereal material, but knowledge about B vitamins in legume-based extrudates is scarce. The results from low moisture extrusion studies are similar to thiamin, riboflavin, and niacin retentions observed in the present study. For example, the reduction of thiamin, riboflavin, and niacin content ranged from 30% to 40% in low moisture extrusion of a mixture of pea and maize [17]. Additionally, in the low moisture extrusion of peas, riboflavin was relatively stable, but the thiamin content decreased by 50% [18]. The moisture contents in these low moisture extrusion experiments were about 10%, which is remarkably lower than the moisture content in high moisture extrusion, which is typically around 50–60%. In addition, these studies were conducted with a single-screw extruder, whereas a twin-screw extruder was used in our experiments. Varying extrusion parameters and their combinations affect, for example, the duration of exposure to high temperatures, and they may also cause differences in vitamin stability. For this reason, studies of vitamin retention in high moisture extrusion are crucial.

As with other B vitamins, the folate contents in the lupin ingredient mixture and extrudates were similar, proving good folate retention in the high moisture extrusion of lupin ingredients (Table 3). Conversely, folate contents in faba bean extrudates were considerably lower (42–67%) than the contents in the respective ingredient mixtures. Thermal treatment of legume material has been observed to lead to loss of folate [39,40]. Thus, the differences in total folate contents between faba bean ingredients and corresponding extrudates suggest that extrusion caused the degradation of folate. In general, folate is susceptible to oxidation, and high temperatures during extrusion may have accelerated the oxidation of folate in the production of faba bean extrudates. However, the oxidative stabilities of folate vitamers differ [39]. Accordingly, we observed different retentions of folate vitamers. The contents of reduced and intermediate forms of folate (H_4_folate, 5-CH_3_-H_4_folate, 5-HCO-H_4_folate, and 5,10-CH^+^-H_4_folate) decreased in the production of faba bean extrudates, whereas the content of oxidised vitamer (10-HCO-PGA) was either retained or even slightly increased. These results support the explanation of folate oxidation in the high moisture extrusion of faba bean ingredients.

Despite the instability of folate, we observed good folate retention in lupin ingredients. Similarly, Ramos Diaz et al. [19] and Gulati and Rose [41] reported that the greatest folate reduction was only around 20% in the low moisture extrusion of corn-based material containing lupin and Great Northern bean flour, respectively. Nevertheless, folate retention was clearly different in faba bean and lupin extrudates in the present study, indicating that the stability of folate in extrusion depended on legume species. In previous studies, folate stability in processing has also been observed to vary among different plant species. Dang et al. [42] reported that folate retentions in soaking, boiling, and pressure cooking were better in chickpeas than in peas. Likewise, Motta et al. [43] determined varying folate stabilities in the boiling and steaming of pseudo-cereals.

The limited extractability of folate in certain matrices is another potential explanation for differences in the folate contents of faba bean ingredient mixtures and corresponding extrudates. Theoretically, processing could have affected the extractability of folate if the compounds were bound to the structures formed in the extrusion process. However, Liu et al. [31] previously determined good recovery of folate in faba bean flour analysed with the same method as used in this study. Overall, the retention of folate in extrudates and its liberation should be studied in the future.

### 3.5. Extrudates as Sources of B Vitamins

Based on the recommended vitamin intakes in the Nordic Nutrition Recommendations 2023 [8], a portion of 100 g of faba bean and lupin extrudates in fresh weight (FW) could contribute to intake as follows: thiamin 14–39% (as contents in extrudates converted to thiamin), riboflavin 5–15%, niacin 3–8% (without contribution of tryptophan), and folate 2–17%. Consequently, the extrudates produced in this study were good sources of thiamin, and some of them could contribute a notable amount of riboflavin to daily intake. Conversely, extrudates did not seem to provide much of the niacin vitamers. However, as protein-rich foods, legume-based extrudates may also contribute to niacin intake through the endogenous conversion from tryptophan [8]. Lupin extrudates were good sources of folate, but faba bean extrudates provided 5% or less of the recommended folate intake.

Meat and dairy products are important dietary sources of thiamin, riboflavin, and niacin in Nordic and Baltic diets [8]. In comparison to the values reported in meat, thiamin and riboflavin contents in extrudates were at the same levels, but niacin contents were markedly lower. In contrast, the folate content in extrudates was generally higher than in meat. On average, the thiamin content in extrudates was 0.3 mg/100 g FW, the riboflavin content was 0.1 mg/100 g FW, and the niacin content was 0.8 mg/100 g FW. Faba bean extrudates contained approximately 12 µg/100 g FW of folate, whereas the content in lupin extrudates was 50 µg/100 g FW. The thiamin content in various raw meat cuts has been reported to range from 0.01 mg/100 g FW to 0.9 mg/100 g FW, the riboflavin content from 0.02 mg/100 g FW to 0.2 mg/100 g FW, and the niacin content from 4.2 mg/100 g FW to 9.6 mg/100 g FW [5,44,45]. Vahteristo et al. [46] determined only around 1–3 µg/100 g FW of folate in various meat cuts and products, but the folate content in chicken was about 15 µg/100 g FW. These reported values are mainly for raw meat, but the vitamin contents may be lower in cooked meat. For example, Lombardi-Boccia et al. [44] observed that cooking decreased vitamin B contents in meat. Likewise, it should be noted that the extrudates are probably heated before consumption. In addition to possible cooking losses, to further evaluate the legume-based extrudates as sources of B vitamins, the bioaccessibility and bioavailability of the vitamins should be studied.

The type of ingredients affected the final vitamin contents and, further, the potential of extrudates to contribute to the intake of B vitamins. Regarding vitamin contents, flours and protein concentrates should be favoured in the production of legume-based products. Conversely, sufficient protein content in extruded materials is needed to achieve a fibrous structure in high-moisture extrusion. Ferawati et al. [14] suggested that the minimum protein content is approximately 50% of solid material; thus, protein isolates provide technological benefits in the production of fibrous plant protein products. In addition, protein isolates have been determined to contain low levels of harmful compounds, whereas these compounds have been observed to accumulate in the protein concentrate [11]. Tuccillo et al. [22] reported that faba bean flour and protein isolate had a milder taste and aftertaste than faba bean protein concentrate. Consequently, choosing between legume flours, protein concentrates, and protein isolates requires balancing nutritional, technological, and sensory advantages.

## 4. Conclusions

In this study, we observed varying vitamin B contents among different legume ingredients. Considerable contents of thiamin, riboflavin, niacin, and folate were determined in flours and protein concentrates, whereas the contents were generally low in isolates. In general, NAM was the main niacin vitamer in legume samples, and 5-CH_3_-H_4_folate, 5-HCO-H_4_folate, and 5,10-CH^+^-H_4_folate were the most abundant folate vitamers. The simultaneous extraction of thiamin and riboflavin and their UHPLC analysis methods was successfully validated and performed. We emphasise including a purification step in thiamin extraction of faba bean samples to avoid interference in pre-column derivatisation. Overall, legume flours and protein concentrates provide a substantial amount of B vitamins in food applications, whereas protein isolates are rather poor sources.

Retention of thiamin, riboflavin, and niacin was generally excellent in the high moisture extrusion of faba bean and lupin ingredients. Folate retention was also good in lupin samples, but there was a difference in folate contents between faba bean ingredients and the corresponding extrudates, indicating degradation of folate in high moisture extrusion. As a result, the instability of folate in the extrusion of faba bean ingredients should be studied in the future. Regarding both vitamin contents and their retention in high moisture extrusion, legume-based extrudates may have a notable contribution to the daily intake of thiamin, riboflavin, niacin, and folate.

In summary, this study provided new information on thiamin, riboflavin, niacin, and folate contents in legume ingredients and extrudates. Further, the results proved that extrudates containing considerable amounts of legume flours or protein concentrates are promising plant-based sources of B vitamins.

## Figures and Tables

**Table 1:** Ingredients and their mixtures and the parameters used in high moisture extrusion experiments.

Ingredients and Their Mixtures	Ratio of Ingredients in Dry Matter (%)	Feed Water Content (%)	Temperature of Long Cooling Die (°C)	Screw Speed (rpm)
FPC ^1,2^	100	60	80	500
FPI:FPC ^1,2^	30:70	60	80	500
FPI:FF:FPC ^1,2^	30:30:40	60	80	500
FPI:FPC ^1^	30:70	62.5	40 and 80	400
FPI:FPC ^1^	50:50	65	40 and 80	400
(LPI:LPC) + LF	(30:70) + 30	45	40 and 80	400
(LPI:LPC) + LF	(50:50) + 30	50	40, 60 and 80	400

FF: faba bean flour; FPC: faba bean protein concentrate; FPI: faba bean protein isolate; LF: lupin flour; LPC: lupin protein concentrate; LPI: lupin protein isolate. Superscripts indicate different manufacturers: ^1^ Suomen Viljava Ltd.; ^2^ AGT Food and Ingredients Inc.

**Table 2:** Method validation results for the analysis of thiamin and riboflavin.

	Linearity and Sensitivity				Intra-Day Repeatability (RSD%, *n* = 3)		Inter-Day Repeatability (RSD%, *n* = 3 × 3)		Recovery (%, Mean ± Standard Deviation, *n* = 3 × 2)
	Calibration Range (ng/Injection)	R^2^	LOD (ng/g)	LOQ (ng/g)	Retention Time	Peak Area	Retention Time	Peak Area	
Thiamin	0.03–0.61	0.999	2.2	6.6	0.4	0.9	1.5	4.3	85 ± 12
Riboflavin	0.08–1.99	0.995	48	144	0.2	0.4	1.9	2.2	91 ± 7

LOD: limit of detection, LOQ: limit of quantification.

**Table 3:** Thiamin, riboflavin, niacin, and folate contents (µg/g DM; ng/g DM for folate) in ingredients, their mixtures, and corresponding extrudates. A significant reduction (%; *p* < 0.05, *n* = 3) of vitamin contents in extrusion is presented in parentheses.

Ingredient (Ratio of Solids %); Extrudate (Extrusion Conditions) ^a^	Thiamin	Riboflavin	Niacin	Folate
FPC-1 (100%)	10.6 ± 0.2	3.3 ± 0.1	21.7 ± 1.5	555 ± 26
Extrudate (60/80/500)	8.6 ± 0.1 * (−19%)	3.2 ± 0.02 * (−3%)	20.6 ± 0.3	212 ± 60 * (−62%)
FPC-2 (100%)	12.6 ± 0.2	6.1 ± 0.2	26.1 ± 1.1	897 ± 75
Extrudate (60/80/500)	11.3 ± 0.1 * (−10%)	5.9 ± 0.5	27.5 ± 0.6	299 ± 11 * (−67%)
FPI:FPC-1 (30:70%)	7.4 ± 0.2	2.7 ± 0.1	14.7 ± 0.8	380 ± 6
Extrudate (60/80/500)	7.4 ± 0.01	3.0 ± 0.2	15.4 ± 0.1	156 ± 6 * (−59%)
FPI:FPC-2 (30:70%)	9.7 ± 0.2	4.7 ± 0.3	19.1 ± 0.5	654 ± 102
Extrudate (60/80/500)	7.9 ± 0.3 * (−19%)	4.6 ± 0.2	18.8 ± 0.4	306 ± 10 * (−53%)
FPI:FF:FPC-1 (30:30:40%)	6.7 ± 0.1	2.6 ± 0.1	13.5 ± 0.1	452 ± 32
Extrudate (60/80/500)	4.8 ± 0.2 * (−28%)	2.5 ± 0.2	13.3 ± 0.5	175 ± 26 * (−61%)
FPI:FF:FPC-2 (30:30:40%)	6.7 ± 0.2	4.2 ± 0.04	17.2 ± 0.7	756 ± 168
Extrudate (60/80/500)	5.7 ± 0.2 * (−13%)	3.6 ± 0.01 * (−14%)	18.2 ± 0.6	296 ± 14 * (−61%)
FPI:FPC (30:70%)	6.7 ± 0.4	2.7 ± 0.1	27.1 ± 1.2	773 ± 79
Extrudate (62.5/40/400)	6.6 ± 0.3	2.7 ± 0.2	26.6 ± 1.4	433 ± 43 * (−44%)
Extrudate (62.5/80/400)	6.5 ± 1.0	2.7 ± 0.2	25.4 ± 0.6	449 ± 47 * (−42%)
FPI:FPC (50:50%)	6.2 ± 0.1	2.3 ± 0.1	21.1 ± 0.3	724 ± 157
Extrudate (65/40/400)	5.9 ± 0.6	2.4 ± 0.03 *	20.3 ± 1.4	305 ± 68 * (−58%)
Extrudate (65/80/400)	5.7 ± 0.07 * (−8%)	2.2 ± 0.1	19.7 ± 0.4 * (−6%)	388 ± 115 * (−46%)
[LPI:LPC] + LF ([30:70] + 30%)	3.3 ± 0.04	1.6 ± 0.01	15.6 ± 0.2	912 ± 93
Extrudate (45/40/400)	4.1 ± 0.2 *	2.0 ± 0.1 *	15.4 ± 0.3	887 ± 55
Extrudate (45/80/400)	4.4 ± 0.2 *	2.1 ± 0.1 *	15.2 ± 0.3	1000 ± 0.2
[LPI:LPC] + LF ([50:50] + 30%)	3.8 ± 0.2	1.8 ± 0.1	15.3 ± 0.3	959 ± 14
Extrudate (50/40/400)	4.4 ± 0.3	2.1 ± 0.1	13.7 ± 0.9	967 ± 182
Extrudate (50/60/400)	4.6 ± 0.02 *	2.2 ± 0.01 *	13.4 ± 0.3 * (−12%)	986 ± 72
Extrudate (50/80/400)	4.5 ± 0.3 *	2.1 ± 0.1 *	12.8 ± 0.4 * (−16%)	930 ± 74

Statistically significant differences in vitamin contents between ingredient mixtures and corresponding extrudates are marked with * (*p* < 0.05). FF: faba bean flour; FPC: faba bean protein concentrate; FPI: faba bean protein isolate; LF: lupin flour; LPC: lupin protein concentrate; LPI: lupin protein isolate. Numbers 1 and 2 indicate different manufacturers. ^a^ Water content (%); temperature of long cooling die (°C); screw speed (rpm).

## Data Availability

The original contributions presented in the study are included in the article, further inquiries can be directed to the corresponding author.
